# Intrinsic Factors Influencing the Infection by Helminth Parasites in Horses under an Oceanic Climate Area (NW Spain)

**DOI:** 10.1155/2009/616173

**Published:** 2009-04-08

**Authors:** I. Francisco, M. Arias, F. J. Cortiñas, R. Francisco, E. Mochales, V. Dacal, J. L. Suárez, J. Uriarte, P. Morrondo, R. Sánchez-Andrade, P. Díez-Baños, A. Paz-Silva

**Affiliations:** ^1^Animal Pathology Department, Epidemiology, Zoonoses and Parasitic Diseases, Veterinary Faculty, University of Santiago de Compostela, 27002-Lugo, Spain; ^2^Centro de Investigación y Tecnología Agroalimentaria de Aragón (CITA), Animal Health Division, Avda. Montaña, 930 50059 Zaragoza, Spain

## Abstract

A coprological survey to determine the influence of some intrinsic factors (breed, age, and sex) on the infection by helminth parasites in equine livestock (*n* = 418) under an oceanic climate area (NW Spain) was conducted. Faecal samples were individually collected and analyzed by the coprological techniques. The main strongylid genera identified were *Trichonema* and *Cyalocephalus* spp (small strongyles) and *Strongylus* and *Triodontophorus* (large strongyles). The prevalence of gastrointestinal nematode was 89% (95% CI 86, 92) and 1% cestoda (0, 2). The percentage of horses with strongyloid parasites was 89% (86, 92), 11% (8, 14) for *Parascaris*, and 3% (1, 5) for *Oxyuris*. The highest prevalence for ascariosis was observed in the youngest horses (<3 years), for oxyurosis in the >10 years animals, and for strongylosis in the 3–10 years ones. Females were significantly more parasitized than males. A negative correlation between the age and the egg-excretion of ascarids and strongyles was recorded. The autochthonous and the English Pure Blood horses were the most parasitized. We concluded that the infections by helminths, especially the strongyloids, are significantly common in the region, so that greater importance should be given to this situation.

## 1. Introduction

The horse industry is increasing because of its diversity, and involves business, sport, gaming, entertainment, or recreation. The raising of horses plays a significant role, due to the high number of animals for leisure, which has generated several direct jobs, such as those needed for handling and the sanity of the animals.

Nowadays, horses are being used in wild pastures and countries for the forest management, utilizing unwanted vegetation for production. For this reason, this industry deserves the attention of the general public and the governments also. This new situation requires more information about the sanitary status of these animals, and the appropriate measures for ensuring the horses are in an adequate fitness condition. Among the diseases affecting horses, helminth infections are much extended [1].

An oceanic climate (also called marine west coast climate and maritime climate) is the climate typically found along the west coasts at the middle latitudes of all the world's continents and in southeastern Australia. Oceanic climates are characterized by a narrower annual range of temperatures than are encountered in other places at a comparable latitude and do not have the extremely dry summers of Mediterranean climates. Under those conditions, survival of parasitology resistant forms is enhanced, enabling thus the possibility for infection throughout the year.

Several investigations have shown the effect of some factors on the infection by parasites in horses. Osterman Lind et al. [2] and Larsen et al. [3] observed the highest output of strongyle eggs in animals aged 2-3 years from Sweden. Bucknell et al. [4] reported that in Australia cyathostomes were more prevalent in young horses, and *P. equorum* was found exclusively in horses less than 2 years of age, and the highest mean intensity of infection for *Anoplocephala perfoliata* was in those horses. Lyons et al. [5] obtained similar results in equids from Kentucky (USA). Nevertheless, there is a lack of knowledge about the influence of breed or sex on the infection by helminth parasites in horses from oceanic climate areas.

This study reports the effect of various intrinsic factors (breed, age, sex) on the equine infection by helminth parasites. Fecal samples were individually collected form 418 horses in an oceanic climate area (NW Spain) and analyzed by the coprological flotation, sedimentation, and migration techniques. Coprocultures were also done to identify the main strongylid genera affecting the horses.

## 2. Material and Methods

### 2.1. Horses

Between April and September 2007, fecal samples from the rectum of 418 horses from NW Spain were collected. According to the breed, four groups were established (Table 1): Spanish Sport Horse (SSH), Spanish Pure Breed (SPB), English Pure Breed (EPB) and the autochthonous Pura Raza Galega (PRG). By considering the age, horses were divided into three groups: G-1 (<3 years old), G-2 (3–10), and G-3 (>10) (Table 1).

### 2.2. Study Design

Although some interesting information about horses management as hygienic conditions, treatment history, and so forth would be helpful to explain the results, it was not possible to achieve in many cases, so we decided not to include it in the current paper. Many samples were collected from horses under an extensive management, and ask those questions to the owners was not possible.

For that reason, the impossibility for ensuring the collection of all data belonging to the total population limited us to the analysis of the factors possibly involved in the infection of equines by helminth parasites. The factors we analyzed corresponded to parameters which can be observed and measured for us in all cases.

The influence of climatic conditions on the dynamics of egg expulsion has been reported [6]. Nevertheless, by considering that the main goal in the current work was to gain more information about the presence of helminth infection in horses and the difficulty in taking more than one fecal sample from each animal in several cases, the data presented here must be comprehended as a coprological survey.

### 2.3. Coprological Analysis

The observation of parasitic forms in the feces of the horses was evaluated by using the coprological flotation, sedimentation, and migration techniques [7]. Five grams of each fecal sample were processed (in duplicate) by the flotation, sedimentation, and migratory techniques [8], with a sensitivity of 10 eggs per gram of feces (EPG).

The laboratory technician conducting the microscope analysis was also blinded to the study design of the selection of the stool specimens.

### 2.4. Genera Identification

Since it is not possible to distinguish strongyle eggs of different species morphologically, fecal samples are cultured for 10–14 days at 20–25°C to allow the development of L3s, which may be collected by means of the Baermann procedure [6, 9]. Genera were identified according to Lichtenfels [10].

### 2.5. Statistical Analysis

By taking account that the egg elimination is not normally distributed, statistical analysis was done by means of the nonparametric Kruskal-Wallis and Mann-Whitney *U* two-sided tests (*α* = 0.05), and significant differences were considered when *p* < .05. The descriptive parameters were the EPG quartiles 1, 2, and 3. All tests were done using SPSS for Windows (14.1).

Prevalences were expressed as the percentage and the 95% Confidence Interval (CI).

The existence of correlation among the different parameters was assessed by using nonparametric Spearman's rank correlation test.

## 3. Results

### 3.1. Identification of Helminth Parasites

Eggs from gastrointestinal nematoda and cestoda were observed in the feces of the horses. No eggs from trematoda or lungworm larvae were obtained.

The analysis of the gastrointestinal eggs showed that the horses were parasitized by *Parascaris equorum*, Strongyles, and *Oxyuris equi*. By means of the coprocultures, we identified *Cyathostominea* and *Gyalocephalus* spp (small strongyles) and *Strongylus* and *Triodontophorus* (large strongyles).

No differences in the genera of strongyles identified according to breed, age, or sex were recorded.

### 3.2. Prevalence of Helminth Parasites

Eighty nine percent (95% CI 86, 92) of the samples had gastrointestinal eggs and 1% (0, 2) cestoda. The prevalence of horses passing *Parascaris* eggs by feces was 11% (8, 14), 88% (85, 91) strongyles, and 3% (1, 5) oxyurids.

As drawn in Table 2, the highest prevalence of parascariosis was obtained in the youngest animals, English Pure Blood horses and females. No *Parascaris* eggs were observed in Sport Spanish Horse animals. Significant differences were obtained by considering the age, breed, and sex of the animals.

The prevalence of strongyles was higher in G-1 and G-2, horses belonging to the autochthonous PRG breed and females. No positive cases were recorded in the Sport Spanish horses. Significant differences were observed by taking into account the age and the breed of the animals.

Table 2 reflects that the greatest percentage of horses with oxyurids corresponded to the oldest animals (G-3), English Pure Blood, and females. No eggs were observed in males or in SSH animals.

Finally, the highest prevalence of horses passing cestode eggs by feces was obtained in the equids from the G-2 (3–10 years), autochthonous (PRG), and females, although these differences were not significant.

Spearman's rank test showed a statistical negative correlation between the age of the horses and the prevalence of ascarids and strongyles (*r* = −0.141, *p* = 0.005 and *r* = −0.143, *p* = 0.004, resp.). Likewise, a weak and positive correlation between age and prevalence of pinworms was matched (*r* = 0.113, 0.023).

### 3.3. Intensity of Infection

The results of the egg-excretion are summarized in Table 3. The highest number of *Parascaris* eggs was observed in the foals, autochthonous horses (PRG), and males. The Kruskal-Wallis test showed significant differences according to the age of the animals.

As drawing in Table 3, there were reached differences in the egg excretion of small strongyles regarding the breed and sex of the horses. The greatest numbers were observed in the English Pure Blood animals and in females, whereas no elimination was observed in Spanish Sport Horses.

No differences were obtained in the egg elimination of oxyurids and cestoda by considering the age, breed, or sex of the horses.

By means of Spearman's rank test, a statistical negative correlation between the age of the horses and the elimination of ascarids and strongyles eggs was proved (*r* = −0.445, *p* = 0.001 and *r* = −0.167, *p* = 0.001, resp.).

## 4. Discussion

Worldwide, horses are exposed to helminth parasites from different groups resulting in significant morbidity and mortality [11]. The most common helminths of horses include the small and large strongyles, along with tapeworms, ascarids, pinworms, and the lungworms [12]. In the current study, we proved the existence of infection by ascarids, strongyles (small and large) and pinworms in horses from NW Spain, which agrees with data reported by Bucknell et al. [4] in Australia, Romaniuk et al. [13] in Polonia, and Pereira and Vianna [1] in Brazil, in horses housed under differing management schemes.

The main helminth parasites observed were strongyles, in agreement with previous investigations in areas with a similar weather [1, 14, 15]. Likewise, a low prevalence was obtained for tapeworms, although the low sensitivity of the fecal examination techniques in detecting cestode eggs could be the explanation to our results [16]. Meana et al. [17] pointed that the coprological methods have a lower likelihood of diagnosing cestode infection when horses have less than 100 tapeworms.

The lack of flukes and coccidia in the current study provides evidence to back up the rarity of infection by this class of parasite [1, 13].

To get more knowledge about the factors influencing infections by helminths in horses, the coprological data were examined by considering the age, breed, and sex of the animals. Only *P. equorum* and strongyles showed any age effect. The values of prevalence and elimination of *P. equi*-eggs were significantly the highest in horses less than 3 years old, which agrees with Mfitilodze and Hutchinson [18] and Bucknell et al. [4].

The percentage of infection by strongyles was significantly greater in the horses less than 10 years old. It has been proved that age does not influence the cyathostome burdens [19], and Klei and Chapman [20] reported that the naturally acquired immunity against small strongyles slowly develops with age, but remains incomplete. This could be the explanation to the finding of a negative correlation between the age and the infection by ascarids and strongyles.

The conditions of housing and management are usually related to the horse breed. This explains why the autochthonous Pura Raza Galega horses, maintained under extensive forest conditions and receiving an inadequate keeping, attained the highest prevalence of infection by ascarids and strongyles. In addition, no helminth parasites were observed in the fecal samples from Spanish Sport horses, and the Spanish Pure Blood ones were few parasitized. Several studies did not match the influence of the horse breed on the helminthosis [18, 21].

It has been shown that helminth infection is ubiquitous in horses with access to pasture. The absence of positive results in the Sport Spanish horses seems to be attributable to a reduced possibility for pasturing and thus to contact to the infective phases (larval 3rd stages for the gastrointestinal nematoda) or to intermediate hosts (oribatids in the case of tapeworms). Another possible explanation could be that these are economically valuable horses, maintained under appropriate hygienic conditions, and receiving periodical veterinary attention. Moreover, the observation of elevated prevalences for gastrointestinal worms in the English Pure Blood and in the Spanish Pure Blood equids was an unexpected result, because of their economic value and the conditions of housing and keeping, similar to the Sport Spanish horses.

Our results showed that sex had influence on the prevalence of ascarids and strongyles, being the females more infected than the males, in agreement with Fikru et al. [21].

This is the first study about the effect of several intrinsic factors on the helminth parasites affecting horses from NW Spain, so the lack of coprological surveys makes more difficult the discussion of the results obtained in the current investigation. It must be taken into account that the presence of seasonal dynamics of egg expulsion influences the results for the intensity of parasitization [6].

The development of coprological surveys provides a very interesting and indispensable tool for knowing the main helminth parasites infecting the horses. In the light of our results we consider that the infections caused by helminths, especially the strongyles, are significantly common in the region of study, so that greater importance should be given to this situation. The elevated prevalence in the English Pure Blood and autochthonous horses underlines the need for the application of appropriate measure to the control of helminth parasites. More information is needed to know the applied ways for the control of parasites in equids, in particular that related to chemoprophylaxis.

## Figures and Tables

**Table 1:** Distribution of the fecal samples from horses under an oceanic climate area (NW Spain) according to their breed, sex, and age.

	Female				Male				Total

Breed	Age (years)								
	<3	3–10	>10	Total	<3	3–10	>10	Total
CDE	4	8	7	19	3	4	2	9	28
PRE	1	6	5	12	3	2	5	10	22
PSI	15	40	20	75	3	5	0	8	83
PRG	40	129	55	224	16	37	8	61	285
Total	60	183	87	330	25	48	15	88	418

**Table 2:** Prevalences of helminth parasites in 418 horses from an oceanic climate area (NW Spain). Results are expressed as the percentage and the 95% Confidence Interval (CI). Spanish Sport Horse (SSH), Spanish Pure Breed (SPB), English Pure Breed (EPB), autochthonous Pura Raza Galega (PRG).

		Ascarids	Strongyles	Oxyurids	Cestoda
Age	<3	22	93	1	0
		(18, 26)	(90, 96)	(0, 2)	
	3–10	6	91	2	2
		(4, 8)	(88, 94)	(1, 3)	(1, 3)
	>10	12	81	6	1
		(9, 15)	(77, 85)	(4, 8)	(0, 2)
	*χ* ^2^	15.842	8.732	5.584	2.228
	*p*	0.001	0.0013	0.061	0.328

Breed	SSH	0	0	0	0
	SPB	5	19	0	0
		(3, 7)	(15, 23)		
	EPB	34	98	5	1
		(29, 39)	(97, 99)	(3, 7)	(0, 2)
	PRG	5	100	2	2
		(3, 7)		(1, 3)	(1, 3)
	*χ* ^2^	64.960	366.936	2.87	0.976
	*p*	0.001	0.001	0.412	0.807

Sex	Male	12	90	3	2
		(9, 15)	(87, 93)	(1, 5)	(1, 3)
	Female	2	84	0	0
		(1, 3)	(80, 88)		
	*χ* ^2^	7.244	3.333	2.799	1.508
	*p*	0.007	0.068	0.094	0.219

**Table 3:** Helminth eggs per gram of feces (EPG) in 418 horses from an oceanic climate area (NW Spain). Spanish Sport Horse (SSH), Spanish Pure Breed (SPB), English Pure Breed (EPB); autochthonous Pura Raza Galega (PRG). Results are expressed as EPG quartiles 1, 2, and 3 (Q1, Q2, and Q3, resp.).

		Statistics	Ascarids	Strongyles	Oxyurids	Cestoda
Age	<3	Q1	0	100	350	
		Q2	50	400	350	
		Q3	137	775	350	
	3–10	Q1	0	100	112	50
		Q2	0	380	150	50
		Q3	50	750	3225	150
	>10	Q1	0	100	100	50
		Q2	0	200	100	50
		Q3	50	500	325	50
		*χ* ^2^	14.781	10.500	1.560	0.2
		*p*	0.001	0.005	0.458	0.655

Breed	SSH	Q1				
		Q2				
		Q3				
	SPB	Q1		25		
		Q2		50		
		Q3		725		
	EPB	Q1	0	413	100	50
		Q2	0	650	150	50
		Q3	50	1275	150	50
	PRG	Q1	0	100	100	50
		Q2	0	300	225	50
		Q3	100	700	1362	150
		*χ* ^2^	3.450	148.841	0.165	0.2
		*p*	0.327	0.001	0.684	0.655

Sex	Male	Q1		50		
		Q2		150		
		Q3		600		
	Mare	Q1	0	100	100	50
		Q2	0	351	150	50
		Q3	50	750	375	100
		*χ* ^2^	0.364	7.942		
		*p*	0.546	0.005		
